# A Rare Case of Giant Facial Myoepithelioma: Diagnostic Approach and Treatment

**DOI:** 10.7759/cureus.111918

**Published:** 2026-07-01

**Authors:** Najib El Orfi, Marouane Imdary, Youssef Bouhajeb, Mohamed Mehdi El Fakiri, Othmane Benhoummad

**Affiliations:** 1 Otolaryngology - Head and Neck Surgery, Centre Hospitalo-Universitaire (CHU) Mohammed VI, Agadir, MAR; 2 Pathological Anatomy and Cytodiagnostics, Ibn Rochd Pathology Center, Agadir, MAR

**Keywords:** accessory salivary glands, facial mass, inner side of the cheek, myoepithelioma, rare benign tumor

## Abstract

Myoepithelioma is a rare benign tumor of the salivary glands, now recognized as a distinct nosological entity. This article presents the case of a large myoepithelioma that developed in the minor salivary glands of the cheek in a 53-year-old married man. The clinical appearance of the lesion, combined with its extension into the deep spaces of the face, initially suggested a malignant tumor. Biopsies performed on the mass suggested a pleomorphic adenoma, but microscopic and immunohistochemical analysis of the surgical specimen confirmed the myoepithelial origin of the tumor. However, the diagnosis of this type of neoplasm remains difficult due to the wide variability of its clinical, histological, and cytological characteristics. A good understanding of the morphological architectures and specific immunohistochemical profiles described in this study is essential for making an accurate diagnosis and avoiding misinterpretation.

## Introduction

Myoepithelioma is a rare benign salivary gland neoplasm composed almost exclusively of neoplastic myoepithelial cells, with absent or minimal ductal differentiation (generally accounting for less than 5-10% of the tumor) and little or no chondromyxoid stroma, distinguishing it from pleomorphic adenoma. These cells are normally present in salivary glands and constitute an important component of several salivary gland neoplasms, including pleomorphic adenoma, adenoid cystic carcinoma, epithelial-myoepithelial carcinoma, basal cell adenoma, and myoepithelial carcinoma. The morphological features of myoepithelioma can vary considerably, reflecting the diverse differentiation potential of myoepithelial cells [1].

Myoepithelial cells are specialized contractile cells located between the epithelial cells and the basement membrane of exocrine glands. They contribute to the expulsion of glandular secretions and help maintain the structural integrity of glandular acini. Neoplastic proliferation of these cells is uncommon and may give rise to tumors with highly variable clinical, morphological, and immunohistochemical features, making diagnosis particularly challenging [1].

Myoepithelioma is an uncommon condition, representing less than 1% of all salivary gland tumors. Histologically, it may exhibit different growth patterns, including solid, myxoid, or reticular arrangements. The tumor cells themselves can display a wide range of cytological appearances, including spindle-shaped, plasmacytoid, hyaline, clear, or epithelioid forms, further contributing to diagnostic complexity [2].

In its benign form, myoepithelioma typically presents as a painless, slowly enlarging mass that may evolve over several months or even years before diagnosis. Due to its indolent course and nonspecific clinical features, it can easily be overlooked or misdiagnosed [3].

Because of its rarity and overlapping clinical, radiological, and histopathological features with other salivary gland tumors, myoepithelioma is frequently difficult to diagnose before surgery. Reporting unusual presentations helps improve clinicians' and pathologists' awareness of this entity and may facilitate earlier recognition and appropriate management [3].

In this report, we describe an unusually large myoepithelioma arising from the minor salivary glands of the cheek, characterized by extensive deep facial extension and a discrepancy between the preoperative biopsy findings and the final histopathological diagnosis. We discuss the diagnostic pitfalls, surgical management, and histopathological features of this rare entity [4].

## Case presentation

A 53-year-old married man, working as a fisherman and with no significant past medical history, presented with a long-standing history of left cheek swelling. The onset of symptoms dates back approximately eight years, beginning with the gradual development of a jugal mass. The swelling steadily increased in size over time, with no periods of remission. Notably, it was painless and not associated with trismus, facial nerve palsy, dyspnea, or dysphagia. The patient remained afebrile throughout the clinical course.

On physical examination, a large, firm, and non-tender swelling was observed in the left cheek. The mass showed no signs of inflammation and caused displacement of adjacent anatomical structures, including the lateral wall of the left nasal cavity, as well as the hard and soft palate. It also extended to the inner aspect of the cheek (Figure 1).

**Figure 1:**
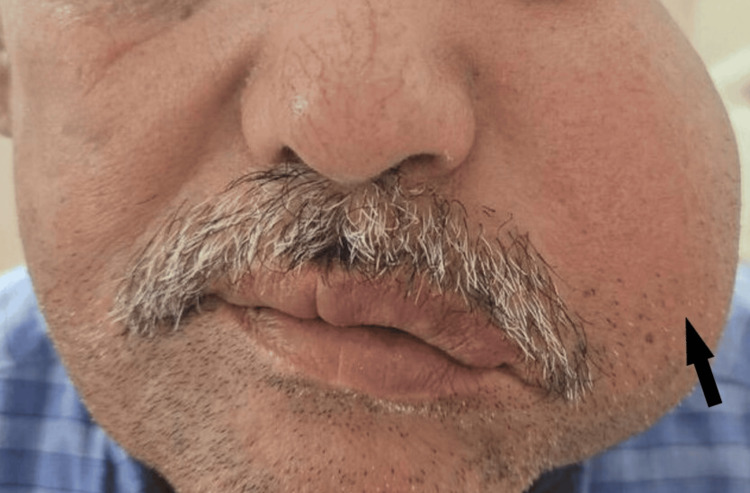
Clinical appearance of the large mass (black arrow) on the patient's cheek, as seen in a frontal view, showing facial asymmetry

A CT scan revealed a locally advanced left jugal mass extending into the infratemporal fossa, with associated osteolytic lesions involving the left wing of the sphenoid bone (Figure 2). Further evaluation with MRI demonstrated a large tumoral process involving the left hemiface, centered on the maxillary and zygomatic bones. The lesion was locally advanced, occupying the lower part of the temporal fossa, the infratemporal fossa, and the pterygopalatine fossa and extending into the submandibular space. It had slightly irregular borders, with a T1 iso-signal, a heterogeneous T2 signal, and areas of T2 hypersignal, as well as diffusion hypersignal with low ADC, and it enhanced intensely and heterogeneously after gadolinium injection, indicating areas of central necrosis. It currently measures 99 mm in height, 79 mm in transverse diameter, and 95 mm in anteroposterior diameter (Figure 3).

**Figure 2:**
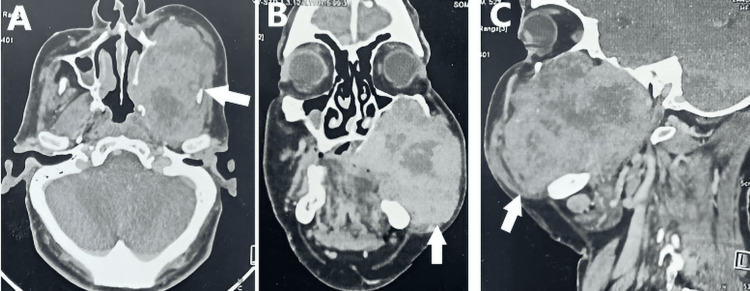
CT scan showing a large tumor mass (white arrows) on the left side of the face A: axial view, B: coronal view, C: sagittal view CT: computed tomography

**Figure 3:**
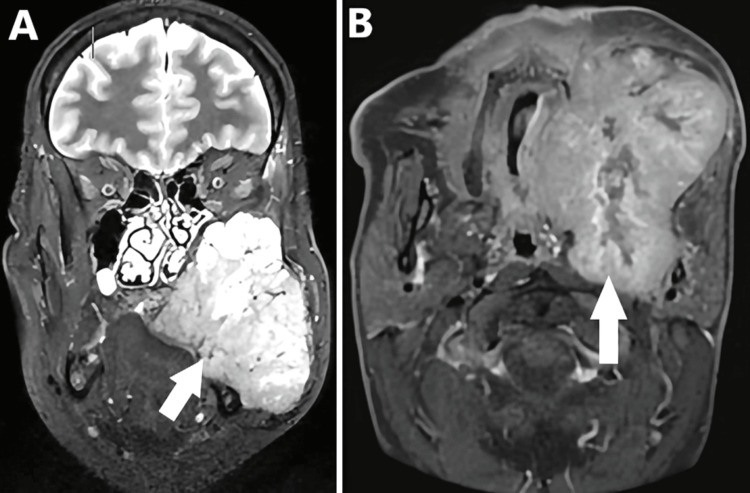
Facial MRI showing a locally advanced mass (white arrows) on the left side of the face A: T2-weighted axial section, B: T1-weighted coronal section MRI: magnetic resonance imaging

The radiological findings suggested an aggressive lesion, given its large size, extension into the infratemporal and pterygopalatine fossae, displacement of adjacent structures, and associated erosion of the sphenoid bone. Although the lesion appeared well circumscribed in several areas, the extensive deep facial extension and osteolytic changes raised concern for a malignant salivary gland neoplasm. Nevertheless, the absence of cervical lymphadenopathy and the relatively homogeneous appearance of the lesion were also compatible with a slowly growing benign process.

A biopsy of the inner cheek was performed under local anesthesia, revealing a pleomorphic adenoma involving the minor salivary glands of the buccal mucosa. A deeper biopsy was then performed to confirm the benign nature of the tumor, which once again confirmed that it was a pleomorphic adenoma (Figure 4).

**Figure 4:**
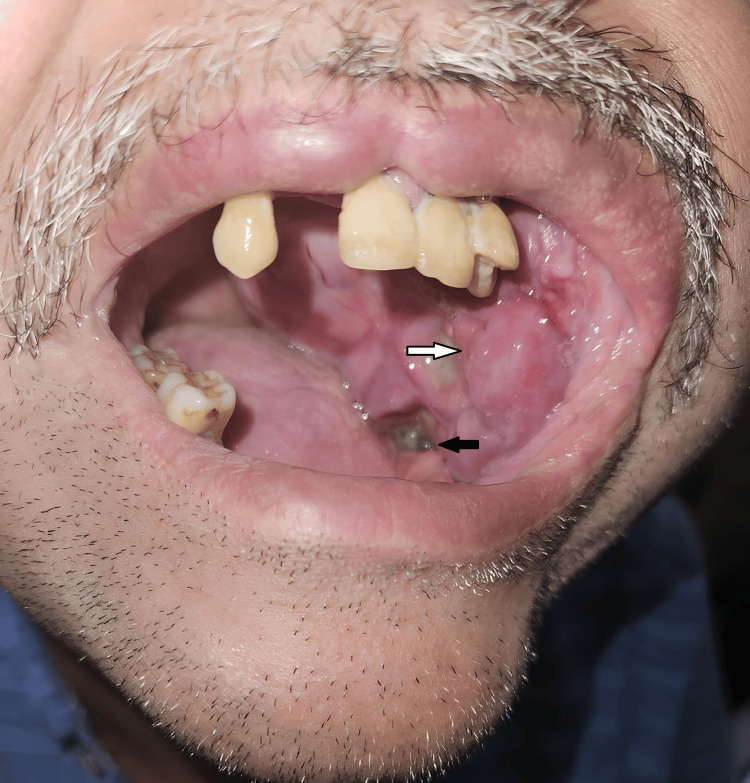
Image of the inner side of the cheek showing the mass (white arrow) and biopsy site (black arrow)

The mass was then surgically removed via a parotidectomy approach, which allowed for precise and controlled access to the affected area. This procedure required meticulous dissection to identify and protect critical anatomical structures, particularly the facial nerve. Preserving this nerve was an absolute priority, both to prevent functional sequelae in the patient and to ensure the success of the surgical procedure.

A standard S-shaped parotidectomy incision was made, extending from the preauricular region down into the neck. A superficial cervicofacial flap was elevated up to the anterior border of the mass, within the plane between the superficial musculoaponeurotic system and the parotid fascia. Dissection was initiated using a cold scalpel and then continued with electrocautery. Throughout the procedure, the assistant carefully monitored for muscle contractions to help prevent facial nerve injury.

The anterior margin of the sternocleidomastoid muscle was identified, dissected, and retracted posteriorly. The posterior belly of the digastric muscle was then exposed, serving as an important deep anatomical landmark for locating the facial nerve. Skeletonization of the external auditory canal cartilage was carried out up to the “pointer,” a key landmark for identifying the principal trunk of the facial nerve. The facial nerve trunk was then carefully identified through meticulous blunt dissection and precise hemostasis. Its branches were dissected anteriorly up to the anterior limit of the mass (Figure 5).

**Figure 5:**
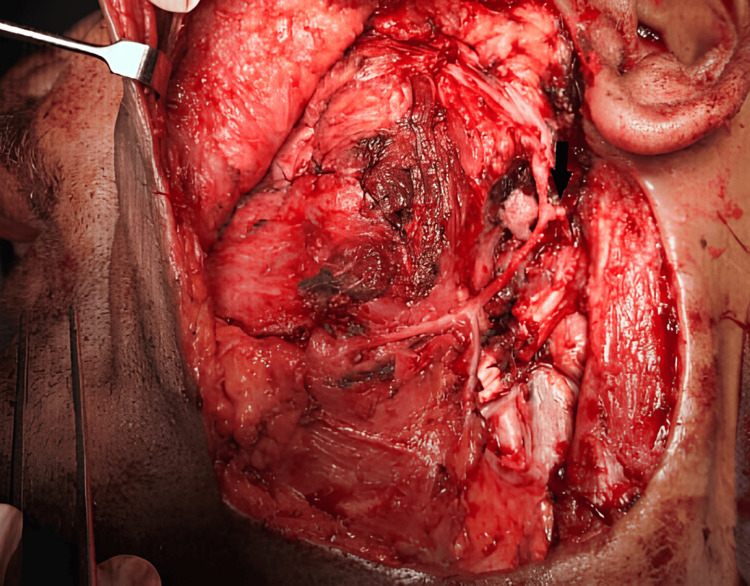
Intraoperative image following dissection of the trunk (white arrow) and branches of the facial nerve

After retracting the branches of the facial nerve backward, we had direct access to the mass, which was located medial to the nerve. The tumor was then carefully dissected; bipolar coagulation was used sparingly to minimize thermal injury to the facial nerve branches (Figures 6-7).

**Figure 6:**
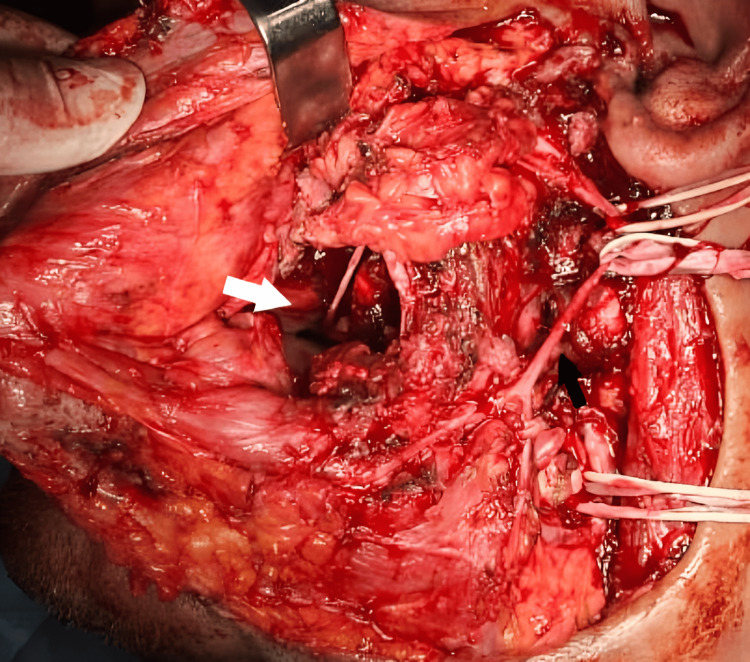
Intraoperative image following total tumor resection showing the cervicofacial branch of the facial nerve retracted backward with a retractor (black arrow) and the site of mass resection with the oral cavity visible through it (white arrow)

**Figure 7:**
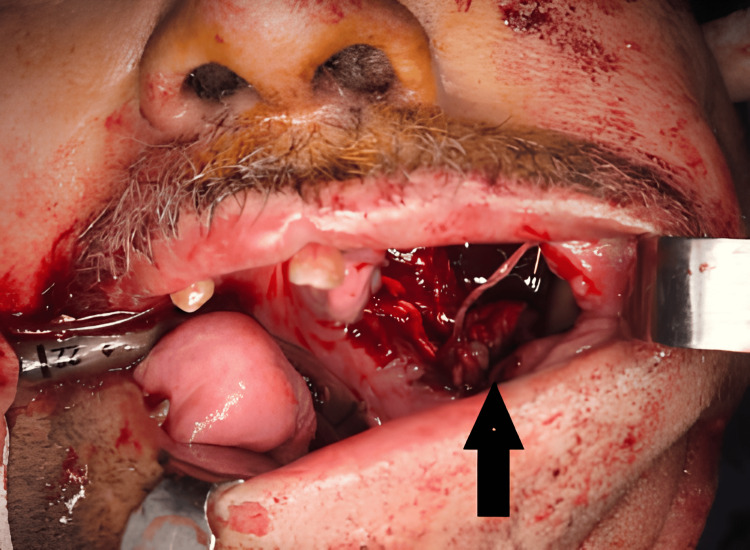
Appearance of the inner surface of the cheek after tumor excision (black arrow)

After excision of the mass, hemostasis was verified; the inner surface of the cheek was then sutured in two layers using absorbable sutures, and the outer surface of the face was sutured layer by layer over a suction Redon drain, which was left in place for 48 hours postoperatively and collected less than 50 mL of blood.

The final histopathological examination of the surgical specimen, supported by immunohistochemical analysis, confirmed the diagnosis of a myoepithelioma with complete excision and clear margins (Figures 8-9). The immunohistochemical study, performed using the Leica Bond-Max platform (Leica Biosystems, Wetzlar, Germany) and the DAKO Autostainer Link 48 platform (Dako A/S, Glostrup, Denmark), revealed the findings shown in Table 1.

**Figure 8:**
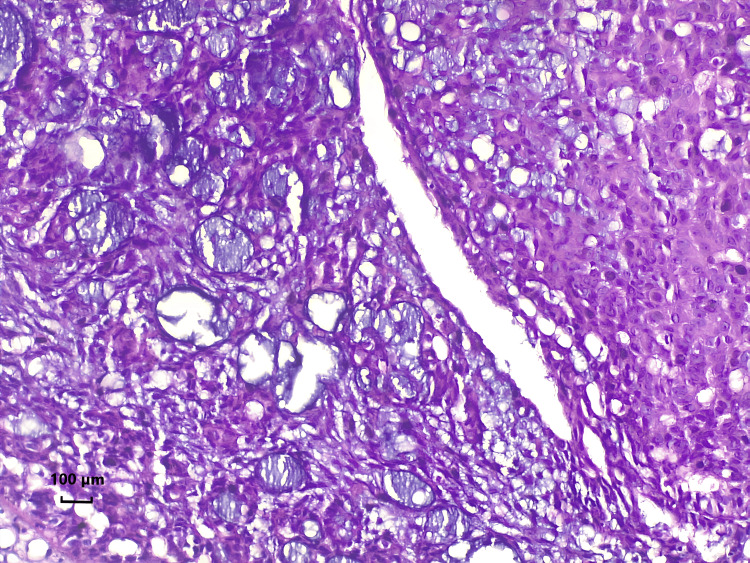
Microscopic view of the histopathological analysis of the surgical specimen (inner surface of the cheek) Magnification at x200, stained with hematoxylin and eosin (H&E); the black scale bar represents 100 micrometers.

**Figure 9:**
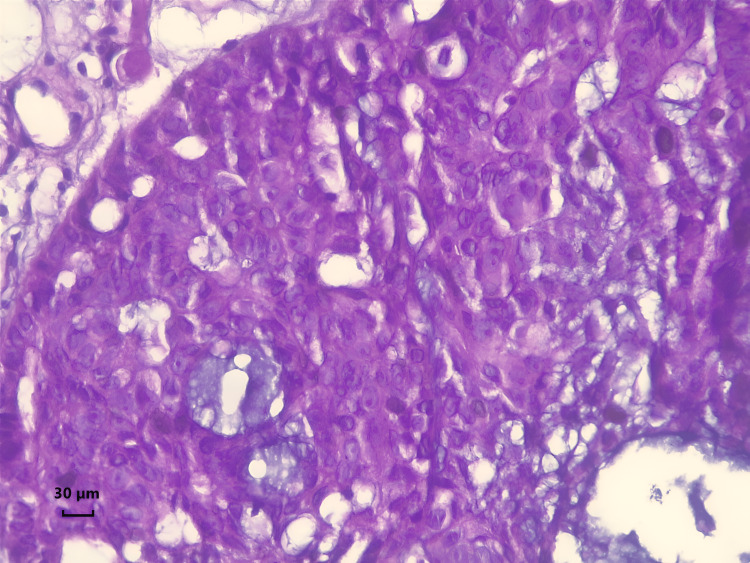
Microscopic view of the histopathological analysis of the surgical specimen (inner surface of the cheek) Magnification at x400, stained with hematoxylin and eosin (H&E); the black scale bar represents 30 micrometers.

**Table 1:** Antibodies tested in the immunohistochemical analysis of the surgical specimen

Antibodies tested	Results
Anti-cytokeratin 7	Positive
Anti-PS100	Positive
Anti-P40	Negative
Anti-DOG1	Negative
Anti-P63	Negative
Anti-smooth muscle actin	Negative
Anti-CD117	Negative
Proliferation index assessed using the anti-Ki-67	Less than 5%

The immunohistochemical profile strongly supported the diagnosis of myoepithelioma. Diffuse positivity for CK7 confirmed epithelial differentiation, while S100 positivity supported myoepithelial differentiation. The absence of DOG1 and CD117 expression, respectively, argued against acinic cell carcinoma and adenoid cystic carcinoma. Negative staining for p40 and p63 excluded squamous and basal/myoepithelial proliferations with ductal differentiation. Furthermore, the absence of smooth muscle actin expression is a recognized finding in a subset of myoepitheliomas, reflecting the variable immunophenotype of neoplastic myoepithelial cells. Finally, the low Ki-67 proliferation index (<5%) supported a benign lesion and argued against myoepithelial carcinoma.

The postoperative course was generally uneventful and without major complications, except for the development of mild facial paralysis on the left side; the patient had already been informed of the risk to the facial nerve prior to the procedure. This condition was classified as Grade II according to the House-Brackmann scale, indicating mild weakness with generally preserved facial function. Appropriate management, combining corticosteroid therapy and facial rehabilitation sessions, led to gradual and satisfactory clinical improvement.

At the 6-month follow-up, the patient remained free of local recurrence. Facial nerve function had almost completely recovered, with only minimal residual weakness, and no new functional deficits were observed.

## Discussion

Myoepithelial cells are contractile cells of ectodermal origin, commonly found in a wide range of secretory tissues, including major and minor salivary glands, lacrimal glands, sweat glands, mammary glands, and the prostate. They are among the most frequent cellular components of salivary gland tumors. Among salivary neoplasms that commonly contain myoepithelial cells are pleomorphic adenoma, adenoid cystic carcinoma, and epithelial-myoepithelial carcinoma, which are believed to arise from the intercalated duct. In contrast, tumors composed solely of myoepithelial cells are rare, representing less than 1% of all salivary gland tumors [1].

Most myoepitheliomas arise in the parotid gland, although they can also develop in the submandibular gland or in minor salivary glands of the oral cavity, including the hard and soft palate, lips, cheek, tongue, floor of the mouth, gingiva, and retromolar area. In some cases, they may develop from glands of the upper aerodigestive tract, such as the nasal cavity, nasopharynx, larynx, and even the lungs [2].

Patients are typically over 50 years of age, with no significant sex predilection. The term "myoepithelioma" was first introduced by Sheldon in 1943; however, the precise criteria for classifying a tumor as such remain a matter of debate. The complex and heterogeneous morphological and immunophenotypic features of neoplastic myoepithelial cells have long attracted scientific interest, although published data are sometimes inconsistent [3].

Traditionally, myoepithelioma was classified into three main histological types: plasmacytoid, spindle cell, and mixed forms. However, recent recognition of a broader range of phenotypic and ultrastructural variations in neoplastic myoepithelial cells has expanded the morphological spectrum of this tumor. This expanded classification accounts for the observed diversity of cytohistological architectures, particularly in myoepitheliomatous areas of pleomorphic adenoma [4].

Unlike pleomorphic adenoma, myoepithelioma lacks true ductal epithelial differentiation and typically has only a scant stromal component. Cytogenetic and molecular analyses have concentrated on pleomorphic adenomas, in which recurrent chromosomal abnormalities have been identified, particularly involving regions 8q12 and 12q13-q15. The exact cellular origin of these alterations remains uncertain. In myoepithelioma, cytogenetic analyses have demonstrated involvement of chromosome 12q12 in a translocation with an as-yet-unidentified partner on 1q, as well as non-random deletions affecting regions 9q22.1-q22.3 and 13q12-q22. These findings suggest that myoepithelial cells themselves are the source of these genetic alterations and that myoepithelioma shares certain chromosomal abnormalities with a part of pleomorphic adenomas [5].

Immunohistochemically, vimentin and S100 protein (although not expressed in normal myoepithelial cells) are considered sensitive but not specific markers of neoplastic myoepithelium. During neoplastic transformation, myoepithelial cells often lose or alter their smooth muscle phenotype, potentially limiting the usefulness of smooth muscle differentiation markers. Nevertheless, positivity for alpha-smooth muscle actin is observed in most tumors, at least in a subset of cells, whether spindle-shaped or plasmacytoid. Myoepithelial carcinomas consistently express cytokeratins, and the absence of keratin expression argues against this diagnosis [6].

The principal differential diagnosis of myoepithelioma is pleomorphic adenoma. Myoepithelioma is composed exclusively, or almost exclusively, of myoepithelial cells, whereas pleomorphic adenoma contains a variable proportion of these cells, sometimes reaching comparable levels. Pleomorphic adenoma is characterized by the presence of numerous ductal structures, which are sparse or absent in myoepithelioma. Stromal components may appear similar in both tumors, including myxoid or even chondroid areas, although these are typically more prominent in pleomorphic adenoma [7].

Myoepithelioma likely represents one extreme of a biological spectrum that also includes pleomorphic adenoma and certain basal cell adenomas (particularly the non-membranous type). Other differential diagnoses include soft tissue tumors such as leiomyoma, which is negative for S100 protein. Schwannomas, although S100 positive, exhibit characteristic histological features; however, Verocay body-like structures have occasionally been described in myoepitheliomas [8,9].

Although most myoepitheliomas are benign, some may exhibit locally aggressive or even metastatic behavior. Histological features indicative of malignancy include infiltrative growth, necrosis, cytologic atypia, increased mitotic activity, and cellular pleomorphism. It has been proposed that evaluating proliferative activity may help differentiate benign from malignant forms, with a Ki-67 labeling index above 10% suggestive of myoepithelial carcinoma [10].

The prognosis of myoepithelioma is generally favorable, provided that complete surgical excision is achieved, significantly reducing the risk of local recurrence. Appropriate management therefore relies primarily on the quality of the surgical procedure and on regular clinical follow-up to detect any potential recurrence. Radiotherapy is typically reserved for situations in which surgery is not feasible, incomplete, or contraindicated, particularly due to tumor location or the patient’s general condition [11].

## Conclusions

The risk of local recurrence primarily depends on the completeness of surgical excision. Myoepitheliomas should be carefully considered, given their potential for aggressive growth, local invasion, and possible malignant transformation, similar to pleomorphic adenomas. Preoperative biopsy can be a useful tool for lesion identification; however, it remains a limited assessment, particularly in myoepitheliomas, which exhibit morphological heterogeneity.

Myoepitheliomas are generally benign tumors, and the overall recurrence rate is low. Complete surgical resection is typically associated with a favorable prognosis. Nevertheless, cases of late recurrence more than 20 years after initial treatment have been reported, underscoring the importance of long-term, careful clinical follow-up.
